# The gut microbiome in disorders of gut–brain interaction

**DOI:** 10.1080/19490976.2024.2360233

**Published:** 2024-07-01

**Authors:** Narjis Kraimi, Taylor Ross, Julien Pujo, Giada De Palma

**Affiliations:** Farncombe Family Digestive Health Research Institute, McMaster University, Hamilton, Canada

**Keywords:** Microbiome, microbial metabolome, disorders of gut–brain interaction, irritable bowel syndrome, functional dyspepsia, abdominal pain, visceral hypersensitivity, gut function

## Abstract

Functional gastrointestinal disorders (FGIDs), chronic disorders characterized by either abdominal pain, altered intestinal motility, or their combination, have a worldwide prevalence of more than 40% and impose a high socioeconomic burden with a significant decline in quality of life. Recently, FGIDs have been reclassified as disorders of gut–brain interaction (DGBI), reflecting the key role of the gut-brain bidirectional communication in these disorders and their impact on psychological comorbidities. Although, during the past decades, the field of DGBIs has advanced significantly, the molecular mechanisms underlying DGBIs pathogenesis and pathophysiology, and the role of the gut microbiome in these processes are not fully understood. This review aims to discuss the latest body of literature on the complex microbiota-gut-brain interactions and their implications in the pathogenesis of DGBIs. A better understanding of the existing communication pathways between the gut microbiome and the brain holds promise in developing effective therapeutic interventions for DGBIs.

## Introduction

Functional gastrointestinal disorders (FGIDs) are recurrent and chronic gastrointestinal disorders characterized by abdominal pain, intestinal motility alterations, diarrhea, constipation, nausea, bloating, vomiting, or a combination of these symptoms.^1–3^ FGIDs are very frequent with a prevalence of more than 40% worldwide, more common in women than men.^1^ The most common FGIDs are irritable bowel syndrome (IBS) and functional dyspepsia (FD), with both disorders significantly affecting quality of life and global health-care costs.^4–6^ Despite the high prevalence, individual and societal impact of these conditions, their etiology, and pathophysiology remain mostly unclear. Indeed, the absence of organic and structural pathologic changes leads to the struggle in diagnosis and treatment approaches.^7^ The most recent Rome IV criteria re-classified FGIDs as disorders of gut–brain interaction (DGBI), acknowledging the role of the psychological aspects in FGIDs physiopathology, as more than two-third of patients suffer from psychological comorbidities.^2,3,8^ The adoption of this novel definition has played a pivotal role in accelerating the progress of the field of neurogastroenterology, with anxiety, depression, and neuroticism emerging as the most frequently reported psychological comorbidities in these patients.^9^ The gut microbiome, the collection of microorganisms present in the gastrointestinal tract, is a major player in gut–brain interactions^10,11^ and alterations of gut microbiome composition are commonly associated with gastrointestinal disorders.^12–14^ Therefore, this review aims first to cover the latest evidence on the role of the gut-brain axis and the microbiome in DGBI, with specific attention to IBS and FD. Next, our focus will shift toward elucidating the most recent progress in comprehending the mechanisms that contribute to this disturbed microbiome-gut-brain interaction in these disorders. Finally, we will recapitulate the existing treatments and promising novel therapies for DGBI, targeting not only gut microbiome and gut function, but also the brain and the psychological aspects of these disorders.

## The gut microbiome

The gut microbiome, a key regulator of gut–brain interaction, is the collection of bacteria, viruses, archaea, and eukaryotes that reside in the human intestinal environment. After rapid colonization at birth, the human intestinal microbiome changes throughout the first 2 years of life, diversifying and then stabilizing into adulthood.^15,16^ Each adult ultimately develops a distinctive microbiome configuration, which appears to be primarily influenced by stochastic environmental exposures in the past,^17^ and additional factors like diet, antibiotic therapy, and host genetics.^18^ Though the origin of microbiome research is often debated, 19^th^ century European scientists Theodor Escherich, Henry Tissier, Ilya Metchnikov, and Alfred Nissle are believed to have pioneered the field of gut microbiome research in human health and disease.^19–25^ Studies on germ-free, microbiome-depleted, or gnotobiotic animals, have highlighted the central role of the microbiome in normal gut physiology, motility, metabolism, and immune functioning.^26–34^ Indeed, it is now well known that the gut microbiome is beneficial to multiple aspects of host function, from host-metabolism, biochemical regulation of processes such as gluconeogenesis, lipogenesis, and cholesterol synthesis,^35^ to healthy aging.^36,37^ This also applies to the gut function, including motility, permeability, and visceral sensitivity. We have recently shown that the gut microbiota drives the physiological development of small intestinal transit via Toll-like receptor (TLR) signaling and small intestinal vasoactive intestinal polypeptide (VIP) regulation of cholinergic nerves.^38^ The microbiota is also key to the development of a normal perception of inflammatory, mechanical, and visceral pain.^39–45^ We have found that the absence of gut microbiota increases visceral sensitivity to colorectal distention through elevated production of calcitonin gene-related peptide (CGRP) by dorsal root ganglia (DRG) neurons in a sex-dependent manner.^46^ Besides the use of gnotobiotic mouse models, the incorporation of modern techniques such as whole genome and targeted metagenomic sequencing,^47,48^ as well as the use of ingestible sampling devices,^49,50^ have aided our understanding of gut microbial influence on health and disease.

Diet is one major factor affecting microbial community structure and function. However, different diets are found to be associated with different beneficial health effects depending on the person.^51^ It has been shown that the same food can elicit divergent responses in different individuals, and the use of personalized nutrition versus a “one size fits all” approach has been widely advocated.^52,53^

Environmental lifestyle is another factor that heavily influences intestinal microbiota composition in multiple ways. Factors including living conditions, antibiotic and medication use, geographical region, lifestyle choices, number of household members, and even pet ownership can contribute to the composition of human microbiota.^54,56^ Alcohol and tobacco consumption as well have been shown to affect microbiota composition and diversity, disrupting the gut microbiome.^57,58^ Very recently, it has been proposed that social microbial transmission, occurring through social interactions and relationships, also plays an important role shaping host health.^59^ Indeed, the authors speculate that socially transmissible commensals and mutualists may modify disease risk for both communicable and non-communicable diseases.^59^ Thus, while it is evident that the gut microbiome is crucial to gut health and homeostasis, we should also remember that it does not live in isolation within the GI tract. Instead, it is in constant “contact” with its host, influencing its symbiont not only locally, but distally as well.

## Microbiota gut-brain axis

The idea of a gut-brain axis dates far back in time within ancient Greece.^60,61^ Studies of gastric acid secretion in human subjects with gastric fistulas conducted in the early 1800s by Beaumont first, and by Wolf and Wolff later, as well as the studies conducted on dogs by Pavlov, scientifically consolidated the concept that human emotions affect host physiology (for a historical overview, see Wolf (1981)).^60^ The gut-brain axis, defined as the bidirectional communication between the central nervous system and the digestive tract, has been linked to the emotional and cognitive centers of the brain with peripheral intestinal functions.^62^ This bidirectional communication system also includes the autonomic nervous system and the hypothalamic- pituitary-adrenal (HPA) axis.^62^ Over the past three decades, scientific knowledge on the gut-brain axis has grown exponentially, and the influence of the gut microbiota on the gut-brain axis has become better understood through the increasing availability of gnotobiotic models. We know today that our microbiome is important for brain structure, development, neurophysiology, and behavior.^10^ This bidirectional communication between the gut, its microbiome, and the brain has also been shown to influence sugar preference,^63,64^ mental health,^65^ mood,^66^ and even decision-making.^67^ Even though the idea that the gut microbiome possibly manipulates host’s behavior to its benefit might be intriguing, the ideas of evolved dependence or local manipulation are likely more realistic,^68^ with the gut microbiome “involuntarily” influencing host’s behavior and brain biochemistry. Clinical and experimental evidence suggests that the gut microbiome, gut, and brain might communicate through multiple direct and indirect pathways, including neural, immunological, endocrine, and metabolic mechanisms.^62^

The vagus nerve, extending from the brain stem to the intestine, is responsible for regulating digestion, vasomotor activity, and the contraction of smooth muscle and glandular secretion.^69^ Additionally, it is considered one of the major communication pathways between gut bacteria and the brain. Vagal nerve afferent fibers reach the brain in the dorsal motor nucleus of the vagus, the area postrema (AP), and the nucleus of the solitary tract (NTS).^70^ Vagal mediation of microbial signals may occur both centrally and peripherally. While more attention has been paid to the NTS as a docking site for vagal afferents, not many studies have looked at the AP, despite its privileged location within the central nervous system (CNS) but outside the blood-brain barrier (BBB), where neurons can be directly influenced by circulating signals.^70^ In the periphery, vagal neurons themselves synapse with intestinal enteroendocrine cells called neuropod cells,^71^ and convey the presence of sugar, regulating sugar preference.^63,72^ Centrally, however, this preference relies on reward neurons, those that mediate motivation and dopamine activity that reside in the right vagus.^73^

Another way of communication between the gut and brain is through modulation of the immune system, which can also be multifaceted. During the last decade, we have learnt that bacteria and bacterial products (peptidoglycan (PGN), lipopolysaccharide (LPS), polysaccharide A (PSA), and short-chain fatty acids (SCFAs) among others) influence the brain and behavior directly or indirectly.^74–78^ Recently, a plethora of studies have shown that bacteria and their by-products can influence brain gene expression, cytokines release, and behavior.^79–82^ These changes can occur through local stimulation of pattern-associated molecular patterns^83,84^ or through the education of immune cells, which then migrate to CNS-associated lymphoid tissue (cervical lymph nodes).^75^ We have recently shown that the establishment of normal behavior in mice depends critically on bacteria-induced activation and migration of intestinal dendritic cells into the brain through the innate immune system and TLR signaling.^85^ Throughout the course of infection, bacteria, endotoxins, and subsequent cytokine-release not only trigger sickness behavior^86,87^ and activate specific neuronal populations,^88–91^ but can also exploit the meningeal neuroimmune barrier, particularly in the case of bacterial meningitis, to facilitate their penetration into the CNS.^92^

While there was some controversy during the past 5 years on whether bacteria themselves can directly access the brain and thus modulate brain biochemistry and behavior,^93–98^ it is essential to learn from the cautionary tale of the extended dispute surrounding the fetal microbiome.^99^ Until robust evidence is presented confirming that bacteria can be found in the brain in non-extreme conditions, a cautious approach is essential.

Finally, bacteria themselves may directly modulate gut-brain communication, as they secrete hormones, catecholamines, neurotransmitters, and lipopeptides,^100–104^ metabolize host hormones,^105^ and modify the structure and the efficacy of commonly used medications.^106–111^ Altogether, these studies highlight how microbial influence on gut-brain communication may occur through multiple routes; thus, the discovery of specific pathways of action will lead to better tailored treatments for microbiome-gut-brain axis disorders.

## Gut brain interactive disorders

**Figure 1.**
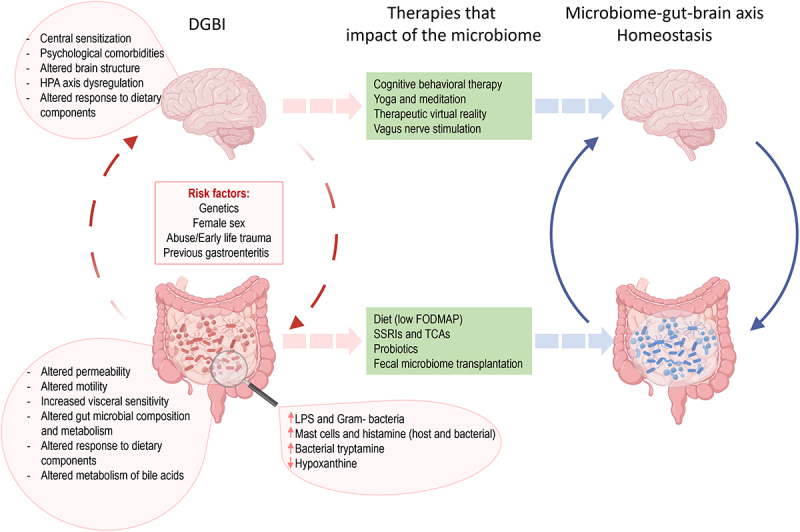
The role of microbiome in disorders of gut brain interaction (DGBI).

The growing recognition of bidirectional interactions between the gut and the brain has greatly contributed to our understanding and management of FGIDs. Indeed, until 2016, FGIDs were defined as a heterogeneous group of chronic or recurrent gastrointestinal conditions that could not be explained by structural or biochemical abnormalities.^7^ Consequently, physicians struggled to identify and understand FGIDs, as they were characterized by the absence of demonstrable anatomic, inflammatory, immunologic, neoplastic, or metabolic response biomarkers, making it difficult to explain patient’s symptoms.^7^ Since 2006, FGIDs were diagnosed and classified using the Rome III criteria which classified adult FGIDs into six categories: esophageal, gastroduodenal, bowel, functional abdominal pain syndrome, functional gallbladder, sphincter of Oddi, and anorectal.^7^ This classification system was updated by the Rome IV criteria system in 2016 resulting in the following categories: esophageal, gastroduodenal, bowel, centrally mediated disorders of gastrointestinal pain, functional gallbladder disorders, sphincter of Oddi disorders, and anorectal disorders.^2,3^ This latest classification included biological in addition to social and psychological aspects of the pathogenesis, with a new definition of FGIDs as DGBI (Figure 1). Several evidence showed that DGBI can result from the combination of genetic, environmental factors such as antibiotic exposure, infections, and physical or sexual abuse, resulting in synergetic gut and psychological health dysfunctions.^7,112–115^ Importantly, the updated definition acknowledged the crucial role of the gut-brain axis and the bidirectional communication between the enteric nervous system (ENS) and central nervous system (CNS) in DGBI pathophysiology.^116^ While the validity of the Rome criteria has been criticized and debated in the past,^117,118^ recent global population-based studies have demonstrated that the Rome IV criteria is a valid tool to diagnose DGBI.^1,119^

The figure illustrates the current knowledge of the pathophysiology of DGBI and current microbiome-directed therapies. On the left are listed all factors that play a role in DGBI pathogenesis and pathophysiology, while in the center are listed all current therapies that are either directed to modify the microbiome and thus improve symptoms or are directed to improve symptoms and indirectly modify the microbiome.

One of the most common and studied DGBI is IBS, with a worldwide prevalence estimated at 4.1–6.1% (Rome IV), varying significantly depending on cohort country and sex, with a higher prevalence in women than men.^1,120^ IBS is a disorder characterized by abdominal pain and altered gut motor function, where patients often present with enhanced visceral perception, altered gut microbial composition and function,^3,12–14,121–123^ as well as genetic and psychosocial components.^7,112,124–127^ Using the Bristol Stool scale, IBS is classified into four different categories according to stool form and bowel habits: IBS with constipation (IBS-C), IBS with diarrhea (IBS-D), Mixed IBS, and Unsubtyped IBS (IBS-U).^128^ While this classification has been created to guide treatment, it has been deemed insufficient to capture the complexity of IBS, a complex multifactorial DGBI. Indeed, seven distinct IBS subgroups have been identified, depending on the severity of gastrointestinal symptoms, extraintestinal symptoms, and psychological comorbidity,^5,129,130^ all factors that weigh not only on patients’ quality of life but also on health-care utilization and costs.

Another common chronic DGBI with an important impact on patient’s quality of life is FD. Large cohort-based studies reported a prevalence of FD varying from 10% to 30% worldwide.^131,132^ FD putative causes include impaired gastric accommodation to a meal, delayed gastric emptying, duodenal sensitivity to acids and visceral hypersensitivity, often associated with genetic and psychosocial factors.^3,131^ Of note, IBS and FD often manifest with similar symptoms; therefore, these disorders may frequently co-exist. It has been shown that patients with an overlap of IBS and FD reported significantly more gastrointestinal symptoms with a greater impact on quality of life and were more likely to report abnormal anxiety and depression scores.^133,134^

As mentioned earlier, the psychosocial and psychological components are among the key factors involved in the pathophysiology of DGBI. The increasing literature about psychiatric comorbidities in patients with DGBI supports the idea of an altered gut–brain interaction.^9,135–140^ The most common psychiatric comorbidities associated with DGBI include but are not limited to: anxiety occurring in 30–50% of DGBI subjects, depressive disorders, neuroticism,^136,138,141–145^ and sleep disorders.^146^ This is mainly evident in patients with IBS, where many studies revealed increased levels of depression and anxiety in comparison to healthy subjects.^147–149^ Psychological comorbidities have been positively associated with higher IBS symptom severity.^150^ A meta-analysis review of 73 cohort studies found that patients with IBS present with three times more risk of developing depression or anxiety when compared to healthy controls.^147^ Similarly, in 2023, an internet survey of the Rome Foundation Global Epidemiology Study on patients from 26 countries worldwide found that psychological comorbidities were reported in 37.5% of the participants and that those participants had 4.45 times higher odds to meet criteria for at least one DGBI.^138^ In line with these findings, a community telephone survey reported a fivefold higher risk of generalized anxiety disorder in responders with diagnosed IBS in comparison to non-IBS individuals.^151^ Indeed, the prevalence of anxiety and depression comorbidities increases with the number of coexistent DGBI, as well as the severity and frequency of gastrointestinal symptoms.^12^ Interestingly, anxiety, but not depression, has been associated with FD,^152,153^ while both depression and anxiety disorders have been associated with functional constipation (FC), another common DGBI with a prevalence rate of 9.5% worldwide in adults and children.^154–158^ Of interest, IBS was shown to share extensive genetic traits with anxiety, depression, and neuroticism,^126,159^ pointing toward the bidirectional link between IBS and psychological conditions.

Hence, it is now evident and widely acknowledged that there is a substantial association between DGBI and psychological health. This recognition has helped to understand the existence of a possible causal link between the two components. However, the direction of the causality remains a “chicken and egg” question, with uncertainty on whether psychological conditions are the cause or the consequence of gut dysfunction and symptoms. Few studies have approached this question and found that psychological comorbidities often precede the development of IBS symptoms.^160,161^ A population-based study conducted in Sweden on FD patients reported that anxiety level at baseline predicted FD 10 years later using the Rome III criteria.^161^ Another study involving 4966 patients with IBS, FD, or FC in the United Kingdom indicated that two-thirds of the patients were diagnosed with anxiety or mood disorders prior to DGBI onset. In 2012, a 12-year prospective population-based study suggested a bidirectional gut-brain effect in people with IBS and FD, revealing that gut dysfunction influences the development of anxiety and depression, and vice versa.^162^ In this study, the authors found higher levels of depression and anxiety at baseline as predictors of DGBI symptoms at follow-up, but also demonstrated that patients meeting DGBI criteria at baseline showed significantly increased levels of depression and anxiety at follow-up visits.^162^ These findings have been further supported by the same authors in a one-year prospective population-based study.^163^ However, it is important to note that this shorter study revealed a higher subset of people with IBS and FD symptoms before the development of psychological conditions, suggesting a role of gut dysfunction as a primary driver of psychological impairment. While more studies have attempted to answer the question on the DGBI originating in the gut or brain,^164^ we should always remember the importance of the gut microbiome, which has been shown to play a central role in gut-brain axis communication, DGBI pathophysiology, and pathogenesis.

## The role of gut microbiota in DGBI

The gut microbiome evolves in a symbiotic relationship with its host and is well known to play a major role in many vital physiological functions including intestinal barrier integrity, immune system maturity, digestion, metabolic activities, and more recently CNS development. Thus, it is not surprising to see the ever-growing interest in the role of the microbiome in the pathophysiology of DGBI.^11,165,166^

While it has been shown that both the mycobiome^167,168^ and the virome^169–171^ are altered in IBS patients, and may play an important, yet under-investigated role, it is still very difficult to disentangle the fluctuations of these populations from those of the bacterial microbiome.^168^ Interestingly, a recent multi-omics study found the virome to be temporally stable and not affected by symptomatic flares in IBS patients but to vary among subsets of IBS patients.^171^ In addition, phages can either directly or indirectly (through affecting the bacterial communities) influence host gene expression.^171,172^ As for the mycobiome, it has been shown that *Candida albicans* species abundance is increased in IBS, and that specific *C. albicans* strains may aid in distinguishing subsets of IBS patients.^167^ However, larger studies that investigate further the role of both gut virome and mycobiome in relation to host and gut bacterial communities are needed.

Post-infectious (PI) IBS is a great example of the involvement of gut microbiome in the pathogenesis of DGBI, as it is characterized by the development of IBS symptoms following infectious gastroenteritis.^173,174^ While for PI-IBS the trigger of symptoms has been quite clear for several decades,^175–177^ the underlying mechanisms are still poorly understood. As much as about 9% of IBS cases in the community have been imputed to previous gastrointestinal enteritis.^178^ It has been suggested that a synergistic interaction occurs between intestinal inflammation and alterations of gut microbiota, stemming from the infection.^178^ In addition, the microbiome might also confer different susceptibility to infection.^178^ Indeed, mechanistic studies have provided evidence to show how infectious gastroenteritis can alter the function and structure of the gut via activation of mast cells, lymphocytes, enterochromaffin cells, enteric nerves, as well as change intestinal permeability, ultimately leading to DGBI symptoms.^178–181^ While in the case of PI-IBS one would think that the root of the disorder clearly lies in the gut and gut microbiome, increasing evidence also suggests an additional role for psychological factors and stress as major risk factors increasing the severity of infection and susceptibility to develop IBS after gastroenteritis.^182–184^ Female gender and prior exposure to antibiotics as well add to the risk of developing PI-IBS following an infectious gastroenteritis.^178^ Genetic predisposition might also be playing a role in the susceptibility to develop PI-IBS,^185,186^ however more studies are needed to confirm these results. With more than 600 million people recovering from the recent COVID-19 pandemic, caused by SARS-CoV-2^187^ and with evidence that SARS-CoV-2 can infect the gastrointestinal tract,^188^ it is plausible to wonder whether the incidence of DGBI, and particularly PI-IBS might increase significantly in the near future. Indeed, a recent study found that 12 months after COVID-19 infection the prevalence of IBS was higher compared with controls.^189^ IBS risk was increased among patients with a history of allergies, chronic intake of proton pump inhibitors and dyspnea at hospitalization.^189^

Antibiotic exposure, commonly seen after infectious enteritis, is known to induce microbiota alterations and appears to be a risk factor for DGBI.^190,191^ Indeed, various studies demonstrated that the development of new-onset IBS symptoms was associated with previous antibiotic use in adults and infants.^192–195^

Many studies have reported alterations of microbiota composition and diversity in DGBI patients when compared to healthy controls^12–14,196–202^ supporting the role of the gut microbiome in the pathophysiology of DGBI. While the research into the gut microbiome in DGBI has grown exponentially during the past decade, and although some bacterial taxa have been reported as altered in a few studies in IBS patients compared to healthy subjects, a particular DGBI microbiome profile does not seem to exist. IBS patients have been shown to have an increased abundance of *Firmicutes* and a decreased abundance of *Bacteroidetes*, associated with an increased *Firmicutes/Bacteroidetes* ratio.^12,197,199,203^ Similarly, various studies reported increased Clostridia and Clostridiales and decreased Bacteroidia and Bacteroidales as well as a reduction in butyrate-producing and methane-producing bacteria^12,197,199,204^ in IBS microbiota. In 2019, a systematic review explored the evidence associating IBS with specific microbiome compositions^201^ and found that IBS patients had microbiomes enriched in facultative anaerobes such as Enterobacteriaceae, and depleted in Uncultured Clostridiales I, *Faecalibacterium* spp., including *Faecalibacterium prausnitzii* and *Bifidobacterium* spp.^201^ However, one of the most striking findings of this analysis was the lack of consistency between the individual studies.^201^ A previous metaanalysis had similarly shown a down-regulation of *Faecalibacterium prausnitzii and Bifidobacterium* spp but also Lactobacillus spp in IBS patients.^205^ A common finding among many of these studies is the overlap between a subgroup of IBS patients and healthy controls,^198,199,206^ highlighting the heterogeneity of the IBS patient population. While all these studies used stool microbiota, it may well be that the attention should be pointed to other sections of the gastrointestinal tract. While most studies focus on the fecal microbiome as it has a higher bacterial density and is readily accessible, recent research has proposed the small intestine and its microbiome to play a role in IBS and FD.^121,207,208^ Indeed, the small intestine microbiota has been reported to be altered and to contribute to symptoms in a subset of IBS patients.^121^ Symptomatic IBS patients present with significant differences in relative abundance of *Porphyromonas*, *Fusobacterium*, and *Prevotella* associated with a lower alpha diversity, richness, and evenness in the small intestine, when compared with healthy volunteers.^121^ Advanced age, antibiotic use, history of GI surgery, and PPI use were found to significantly contribute to these differences in SI microbiome.^121^ Similarly, alterations of small intestine microbiota composition have been found in the duodenum of FD patients with differences in the relative abundance of *Actinomyces, Prevotella, Veillonella*, and *Streptococcus*, along with correlations between bacterial load and FD symptoms.^207^

Small intestinal bacterial overgrowth (SIBO), defined as a quantitative increase of the small intestinal bacteria, has been suggested to drive gastrointestinal symptoms such as diarrhea, abdominal pain, and bloating in a subset of IBS patients.^121,209^ However, several systematic reviews and meta-analyses that investigated a link between IBS and SIBO found an overall low quality of evidence, mainly due to the clinical heterogeneity of subjects included and the limited sensitivity and specificity of the available diagnostic tests. ^210–212^ This controversy is also reflected in recent studies. Although patients with IBS and more than 10^3^ colony forming unit per mL of duodenal aspirates, enrichment in specific *E. coli* and *Klebsiella* strains, have been shown to have abdominal pain, gas and diarrhea,^209^ another study found no correlation between SIBO and gastrointestinal symptoms, and attributed SIBO to environmental influences such as dietary preferences.^121^

Factors hindering our capacity to find a clear IBS or DGBI bacterial signature include: the lack of consistent methodologies used to assess microbiome composition, the lack of rigorous statistical testing, the cross-sectional nature of most of the data, the geographical variability of data, the omission of dietary information in most studies, the focus on fecal and colonic microbiome alone, and the inherent variability of patients.^201,213^ In addition, the mucosal microbiota may also play an important role in DGBI pathophysiology. However, the same confounding factors discussed above (location of tested sample, methodology used to assess the microbiome, etc …) apply also to those few studies that ventured into looking at the mucosal microbiota of patients with DGBI, particularly IBS.^214–218^ Of note, despite all these confounding factors, few studies have found a common decrease in microbiome richness and diversity in IBS patients and an increase in Proteobacteria, particularly *Pseudomonas* spp.^215,217,218^ In addition, low-grade inflammation has been recently shown to accompany alterations in the mucosal-associated microbiome.^217^ Whether an altered mucosal microbiome induces low-grade inflammation or low-grade inflammation impacts the microbiome remains to be determined. Recent studies have revealed enhanced translocation of live bacteria and mucosal immune activation in colonic biopsies of patients with IBS,^219^ supporting the idea of a compromised intestinal barrier in IBS.^220^ Likewise, our preclinical study using IBS humanized mice reveal a thinning of the mucus layer and bacterial translocation into the lamina propria.^221^ This underscores the significance of mucus layer integrity in averting further immune activation, with the microbiome likely playing a significant role in this process.

Furthermore, the microbial metabolic activity, and not necessarily its structure, may be important in DGBI pathogenesis. The metabolome of IBS patients is unique and characterized by altered bile acids, amino acids, fatty acids, organic acids, histamine, tyramine, decreased degradation of lactose and galactose, and increased fermentation of carbohydrates when compared to healthy individuals.^14,199,200,202,222,223^ In a longitudinal multi-omics study, Mars *et al*. nicely described not only microbiota composition changes in IBS patients, with higher abundance of multiple *Streptococcus* spp. and lower abundance of the phylum Synergistetes compared to healthy controls, but also identified the host-microbial pathway of purine metabolism as an important player in the pathophysiology of IBS.^14^ They identified hypoxanthine that was decreased in IBS likely due to an increased xanthine dehydrogenase/oxidase activity from both the microbiome and the host, suggesting a possible therapeutical target for this subset of IBS patients. In addition, the fecal metabolome of IBS patients has been shown to be able to discriminate between subtypes of IBS.^14,199,202,224,225^

Abnormal bile acid metabolism with increased fecal primary bile acids or idiopathic bile acid malabsorption (BAM) has been reported in about 30% of patients with IBS-D.^226–228^ These patients have a significantly faster colonic transit and an altered microbiome with decreased dihydroxylation capacity and sulfatases that distinguish them from other IBS patients.^199,200,228^ They also present with an upregulation of barrier, immune, and inflammatory markers in the colon, as well as a loss of mucin, pointing toward the detergent and proinflammatory effects of bile acids.^228^

Besides bile acids, subtypes of IBS may also be differentiated by the abundance of fecal Clostridiales and SCFAs.^229^ Further, a recent study reported that while IBS-D and IBS-U patient’s microbiome presents with a decreased capacity to degrade lactose and galactose as well as an increased capacity to produce hydrogen sulfide, IBS-C patient’s microbiome has an elevated capacity to degrade phenylethylamine and synthesize palmitoleate.^223^

Altogether, these data suggest the potential role of the microbial metabolome as a biomarker tool to discriminate DGBI. However, the exploration of microbiome function via the analysis of the fecal metabolome, though initially considered groundbreaking, has clearly produced highly variable results.^230^ This underscores the importance of adopting a standardized longitudinal multi-omics integrative approach.^14,213^ The ultimate objective of this approach is to advance the development of mechanism-based targeted therapeutics and to pinpoint the specific patient cohorts that would benefit from these therapies. To this end, translational animal studies provide the mechanistic insight that clinical trials are not able to offer and have significantly advanced the field throughout the past few years. Furthermore, translational studies can contribute to resolving the question regarding whether modifications in the gut microbiome, encompassing shifts in relative abundance, composition, diversity, and function, are cause or consequences of disturbed gastrointestinal motility, and gastrointestinal dysfunction.

We have shown that the gut microbiome plays a causal and functional role in the pathophysiology of IBS, as transplantation of fecal microbiota from IBS patients into germ-free mice resulted in alterations of gastrointestinal transit, gut barrier function, innate immune responses, and behavior.^231^ In this study, we demonstrated that the metabolism of the microbiome in individuals with IBS is crucial to the model. While the composition of the microbiome was quite similar in mice receiving samples from both healthy individuals and patients with IBS, the serum metabolome was significantly different. Using this model to explore additional microbiota-driven pathogenic mechanisms, we have found that histamine produced by the microbiome can engage the host immune system, in particular mast cells, via activation of histamine 4 receptor (H_4_R) and lead to the development of visceral hypersensitivity in a subset of IBS patients.^221,232^ In addition, we show that targeting either H_4_R or bacterial histamine production through dietary modifications may constitute a novel therapeutic approach for this subset of IBS patients.^221,232^ However, multiple mechanisms likely play a role in IBS pathophysiology and hypersensitivity, as we also found that in a subset of IBS patients that respond to a low FODMAP (Fermentable Oligosaccharides, Disaccharides, Monosaccharides, and Polyols) diet, histamine together with protease signaling, both likely of bacterial origin, modulate nociceptive nerve activity.^233^ Indeed, the role of mast cells and histamine, of host and bacterial sources, is central in pain sensation in DGBI.^123,221,232–235^ Several studies have suggested a mechanistic role of histamine-mediated transient receptor potential channel sensitization in IBS pathophysiology via histamine 1 receptor (H_1_R) activation, resulting in increased visceral pain perception,^181,237–239^ where treatment with an H_1_R antagonist is of moderate efficacy in a subgroup of patients.^237–239^ These translational studies suggest that multiple mechanisms may be at play in the same subset of patients, thus explaining the modest efficacy of certain medications and advocating for the design of combination therapies that target both the microbiome and the host.

Another recent translational study implicated bacterial proteases in the pathophysiology of PI-IBS, revealing high gut proteolytic activity driven by host serine proteases,^240^ confirming previous findings.^241^ This study suggests that a reduction in microbial β-glucuronidase activity may contribute to IBS pathogenesis, as β-glucuronidases released by commensal microbes suppressed host proteolytic activity, thereby protecting the intestinal epithelium.^240^ Aberrant amino acid metabolism has been reported in IBS patients, and a recent mechanistic study demonstrated how tryptamine, a tryptophan-derived monoamine, regulates ion flux across the intestinal epithelium via 5-Hydroxytryptamine_4_ (5-HT_4_) receptor activation, affecting intestinal motility.^242^

All these studies have not only advanced our understanding of the pathophysiology and pathogenesis of IBS, but also revealed several possible targets for the design of novel therapeutic approaches. While the functional role of the gut microbiota in DGBI is well demonstrated, one must remember to consider its interaction with the central nervous system through the microbiota-gut-brain axis as a potential mechanism for both pathogenesis and treatment. In the next section, we will describe the current most used microbiome-directed therapies for the treatment of symptoms in DGBI.

## Current treatments

Within the field of DGBI research, the therapeutic interventions being developed are focusing on symptom relief (Figure 1). Currently, there are no long-term cures available for DGBI, as our understanding of the underlying mechanisms is incomplete. However, therapies directed to the management of bowel symptoms and the psychological aspect of these disorders have progressed. The latest guidelines for treatment of DGBI from the American Gastroenterological Association (AGA) have recommended to involve nonpharmacologic therapies early in the treatment plan, while moving away from opioid prescription for pain relief.^243^ While the emphasis is on non-pharmacological therapies, in 2022 the AGA made the recommendation for some pharmacological agents to be used as treatments for IBS-D, such as eluxadoline, rifaximin, and alosetron,^244^ leaving the use of selective serotonin reuptake inhibitors (SSRIs) and tricyclic antidepressants to the physician’s discretion, given the efficacy for a subset of patients.^244^ A recent randomized, double-blind, placebo-controlled trial found a beneficial effect of low-dose amitriptyline in IBS, suggesting this inexpensive and safe treatment for general practitioners.^245^

As the focus of this review is on the microbiome in DGBI, and thus microbiome-directed therapies for DGBI, we should remember that many of the commonly used medications have an overlooked effect on the microbiome.^106,108^

Bile acid sequestrants, such as cholestyramine, colestipol, and colesevelam, have been used to treat IBS patients with BAD, and while their efficacy needs to be tested with larger clinical trials, it appears that bile acid sequestrants are effective in improving abdominal symptoms and both stool frequency and consistency, without targeting the underlying pathophysiology.^226,246^ Interestingly, clinical response to bile acid sequestrants appears to be associated with compositional and functional alterations in the gut microbiome, highlighting a possible interplay between bile acids and the gut microbiome.^247,248^

While antibiotic exposure has been described as a risk factor for development of DGBI, some antibiotics appear to be helpful in treating existing DGBI.^249^ One of the first antibiotics used in a randomized controlled trial with IBS patients was neomycin which resulted in a greater relief of symptoms in 35% of patients compared to only 11% in placebo subjects.^250^ However, due to its potential side-effect, in particular ototoxicity, the use of neomycin has been limited. Other antibiotics have been shown to successfully improve both IBS gastrointestinal symptoms and SIBO. Among them, we can find norfloxacin^251^ and the more commonly used rifaximin, a broad-spectrum, non-absorbable antibiotic.^252^ Although a recent systematic review and network meta-analysis evaluating pharmacological treatments for IBS-D patients found rifaximin to have limited efficacy in alleviating overall IBS symptoms and abdominal pain, its approval by the Food and Drug Administration (FDA) for treating IBS-D stems from its favorable safety profile and minimal adverse effects.^253^ While the exact mechanisms of action remain unclear, it has been demonstrated that rifaximin treatment is associated with acceleration of ascending colon transit^253^ and changes in gut microbiota composition with specific increase in *Faecalibacterium* abundance correlated to clinical improvement.^254^ Rifaximin has also been suggested to have direct anti-inflammatory activity, effects on microbial mucosal adherence or bacterial virulence,^255^ and may act on the CNS given the neuroactive potential of antibiotics.^256^ Furthermore, rifaximin seems to prevent the development of increased intestinal permeability and visceral hyperalgesia in a model of chronic psychological stress.^257^

Neuromodulators, such as antidepressants, also possess antimicrobial properties.^258^ The SSRI fluoxetine, for example, has been shown to act indirectly as a bacteriostatic agent on the bacterium *Turicibacter sanguinis* that can import serotonin and use it to thrive.^259^ Thus, the efficacy of some anti-depressants in symptoms of DGBI may be uncoupled from their central effects, and instead be solely peripheral or microbiome mediated. Furthermore, besides the microbiome, genetics may also play a role in influencing neuromodulator metabolism and efficacy.^260^ As the microbiome is central to both gut physiology and psychological wellbeing, microbiome-directed therapies have been increasingly advocated for DGBI. Diet, as a major modulator of gut microbial composition and function, is also considered a first-line treatment for patients with DGBI,^261–263^ due to a great proportion of patients often complaining about food triggering or worsening symptoms. While some studies reported a beneficial effect of a gluten-free diet in subsets of patients with IBS,^264,265^ the evidence is against recommending gluten-free diet for patients with IBS.^266,267^ Certainly, it remains unclear whether the reported positive effects were a result of gluten protein withdrawal, the reduction of fermentable fructans, the microbiome’s capability to process gluten antigens and alter their immunogenicity, or potentially the presence of amylase trypsin inhibitors (ATIs) found in wheat.^264,265,267–272^ In addition, our recent study highlights the importance of central mechanisms in a subset of IBS patients with self-perceived gluten sensitivity.^273^ This may also be the case of FODMAPs, which were shown to modulate the activity of several brain areas related to pain, such as the cerebellum, supramarginal gyrus, anterior and midcingulate cortex, insula, and thalamus in IBS patients.^274^ The low FODMAP diet is currently one of the most adopted diet interventions for IBS and has been found to be more effective at reducing IBS symptoms than other dietary approaches.^275^ While insoluble fiber significantly worsens IBS symptoms, soluble fiber has been positively associated with IBS symptom improvements.^261^ Gunn et al. reported that psyllium reduces inulin-related gas production in IBS patients, suggesting the use of soluble and poorly fermented fibers like psyllium as a prebiotic pairing with FODMAP-rich foods, to achieve the reduction of colonic gas and breath hydrogen response.^276^ In healthy individuals, however, a low-fiber diet leads to development of gastrointestinal symptoms as well as changes in intestinal permeability and microbial diversity,^121^ underscoring the importance of individual microbiomes underlying specific dietary responses.

Mechanistic studies have highlighted the role of bacterial LPS, bacterial histamine, and proteases as possible modulators of host responses to diets high in fermentable carbohydrates.^233,277–279^ Indeed, the microbiome has been postulated as a driver for the individual response to a low FODMAP diet, given that the colonic microbiota ferments undigested carbohydrates reaching the large bowel.^263^ Furthermore, host genetics have been implicated in host responses to diet modifications in IBS patients.^125^ For instance, the efficacy of a sucrose and starch-restricted diet or low-FODMAP diet has been recently linked to the dysfunction of the sucrase-isomaltase gene in IBS-D patients.^125^ Thus, all these data highlight the need to contemplate both host microbiome and genotype when considering dietary restrictions in DGBI, particularly when considering a diet as restrictive as the low FODMAP diet.

The use of probiotics for DGBI treatment has gained traction over the last decade. Despite reports of some efficacy of probiotics in IBS,^280^ the latest guidelines for IBS and FD have not endorsed their use for global relief of symptoms or specifically bloating and distension, due to the relatively low quality of evidence.^281,282^ Confounding factors for the assessment of probiotics’ efficacy lie not only in the heterogeneity of the patients’ populations (diagnostic criteria, geographic location, etc …) included in each study, but also in the different probiotic formulations tested (i.e., different preparation and storage, different strains or combination of strains, contained in each formulation, and so on). A recent meta-analysis identifying 82 clinical trials investigating the use of probiotics in IBS determined only 24 to have a relatively low risk of bias using the Cochrane risk of bias tool.^283^ Of note, certain probiotic strains have been recommended to treat global IBS symptoms in children in Italy.^284^ Probiotics might affect central symptoms in DGBI patients rather than directly improve GI symptoms, as evidenced by few studies in both patients and animal models.^285–287^ However, to comprehensively grasp the potential benefits, both at the central and peripheral, of probiotic administration for patients with DGBIs, future large-scale and well-designed randomized controlled clinical trials (RCTs) are necessary.

Another microbiome-directed approach that has engaged a considerable amount of recent research is the fecal microbiota transplantation (FMT), a process that involves the transfer of stool from a healthy donor to a recipient via colonoscopy, nasogastric tube, colonic enema, or capsules; a well-known and highly effective treatment for recurrent *Clostridioides difficile* gastrointestinal infections.^288^ Given the evidence highlighting a central role for the gut microbiota in DGBI pathophysiology, FMT has been proposed as a treatment strategy for IBS. A recently published randomized placebo-controlled trial on patients with treatment-refractory IBS reported an improvement of symptoms when receiving FMT compared to placebo. In this study, patients were followed for several months and the observed beneficial effects of FMT decreased over 1 year, while a second FMT led to a restoration of the effects.^289^ In contrast, long-term efficacy has been reported at 2 and 3 years after a single FMT, with better outcomes, including fewer IBS symptoms and fatigue, and without long-term adverse effects.^290^ However, a recent meta-analysis on the efficacy of FMT in IBS reported no overall advantage of FMT over placebo, although delivery to the small intestine (vs. colon or capsules) appeared to be effective.^291^ Indeed, the authors stressed the need for well-designed RCTs and long-term follow-up registries to ensure the efficacy and safety of FMT. The same conclusions were also reached in another meta-analysis published in 2023 highlighting the overall low quality of evidence for FMT in IBS.^292^ Repeated delivery of FMT to the small intestine appears to yield prolonged relief from symptoms and an enhancement in quality of life, as highlighted by a recent study.^293^ While there appears to be a future for FMT in treating IBS, the specific mechanisms responsible for its effectiveness remain unclear. In addition to considering the microbiomes of both the patient and the donor, other variables such as the patient’s clinical characteristics, diverse administration routes, and the quantity of fecal material administered play a role in FMT clinical outcomes. Recently, the idea that strain engraftment may be correlated with clinical improvement post-FMT has been introduced as an additional factor influencing the success of FMT in IBS and other disorders.^294^

Besides microbiome-directed therapies, the field has also seen a rise in therapies aimed at addressing the central dysfunction and psychological comorbidities of DGBI. Assessing the efficacy of over 40 eligible randomized controlled trials undergoing a form of psychological intervention for IBS, Black *et al*. identified evidence supporting the efficacy of cognitive behavioral therapy (CBT) and gut-directed hypnotherapy.^295^ Indeed, CBT improves IBS symptoms^296^ and its response has been associated with baseline intestinal microbiota and serotonin levels.^297^ These data provide further evidence that not only microbial signals can modulate central processes involved in symptom generation and sensation in IBS, but also that, in a subset of patients, the brain exerts a large influence on the gut microbiome. Therapies focusing on mind–body interactions and stress reduction may also be integrated into the management of DGBI.^298^ For example, in-person and virtual yoga practices have been reported to be effective at reducing symptoms as well as decreasing anxiety, depression, and stress in IBS patients.^299–307^ While psychiatric influence on the gut microbiome has been moderately investigated, there is limited evidence on the effect of yoga and meditation practices on gut microbial composition and function in DGBI.

Over the last few years, therapeutic virtual reality (VR) technology has become a topic of discussion for effective DGBI symptomatic relief, so much so that it has recently become an FDA-recognized area of medicine known as “medical extended reality”.^308^ Using an electronic headset over the eyes, patients immerse their mind into a virtual world that acts as a distraction from noxious peripheral stimuli, creating a therapeutic illusion.^308^ Interestingly, VR has been shown to be effective in treating psychological disorders such as anxiety and depression, which are frequently comorbid with DGBIs and exacerbate symptoms such as abdominal pain.^308^ While VR seemed to be effective for FD,^309^ it has yet to be tested as a treatment for IBS.^310^ Further studies investigating not only the therapeutic benefits of VR on DGBI as well as the impact of this form of therapy on the microbiome are needed.

Finally, given the importance of the vagus nerve in microbiome-gut-brain communication, the use of vagus nerve stimulation (VNS) as a potential therapeutic intervention for DGBI, particularly for dysmotility, inflammation, and pain, has prompted interest.^311,312^ VNS has been approved by the US FDA for epilepsy and pharmaco-resistant depression, but due to its invasive nature, has not been approved for any gastrointestinal disease. However, noninvasive VNS techniques have been recently used to treat abdominal pain and constipation. A recent RCT testing the efficacy of transcutaneous auricular VNS in patients with IBS-C found a significant reduction in abdominal pain, constipation, pro-inflammatory cytokines, and serotonin production, as well as a significant improvement in quality of life.^312^ The results of this study prompted the hypothesis that auricular VNS decreases inflammation by activating the cholinergic anti-inflammatory pathway and, in turn, alleviating visceral pain.^312,313^ This hypothesis is supported by a recent animal study reporting that VNS improves visceral sensitivity, depression, and inflammatory mediators via the a7nAChR-mediated inflammatory pathway in a mouse model of IBS.^314^ Likewise, auricular percutaneous electrical nerve field stimulation (aPENFS), believed to target the vagus nerve, has demonstrated encouraging outcomes in mitigating visceral sensitivity and abdominal pain in three clinical trials involving adolescent IBS patients.^315–317^ Additionally, although one of these trials did not observe a strong microbiome change in pre- versus post-therapy, or between responders and non-responders, the authors showed a higher abundance of *Blautia* in responders compared to non-responders.^316^

These data substantiate the involvement of gut-brain communication through autonomic function and vagus nerve activity in DGBI, underlining the importance of initiating new clinical trials in adult populations with DGBI that comprehensively evaluate all aspects of these disorders, encompassing gut symptoms, psychological comorbidities, and gut microbiome composition and function. Taken together, these discoveries reiterate the significant heterogeneity among DGBI patients concerning both clinical manifestations and responses to treatment.

## Conclusions

The growing body of literature on the microbiota-gut-brain axis and DGBI is aiding our understanding of the mechanisms underlying DGBI pathophysiology. While the pathways involved in the communication between the intestinal microbiota or specific bacterial strains, the gut, and the brain in the context of DGBI are still not fully understood, recent evidence has demonstrated different microbes and their metabolites to play a crucial role in a variety of patient subsets, driving specific symptoms such as abdominal pain or dysmotility. Large-scale longitudinal multi-omics trials, combining the study of both host and microbiome, with a focus on microbiome function, coupled with translational studies, appear to be the recommended approach to generate findings that will help understand specific mechanisms driving symptoms in subsets of patients. These studies will aid the development of new microbiome-targeted diagnostic techniques and therapies, aimed at streamlining patients’ management and treatment, and reduce the overall socioeconomic impact of these challenging disorders.
